# Ultra-Low-Dose CTPA Using Sparse Sampling CT Combined with the U-Net for Deep Learning-Based Artifact Reduction: An Exploratory Study

**DOI:** 10.1007/s10278-025-01639-8

**Published:** 2025-08-27

**Authors:** Andreas Philipp Sauter, Johannes Thalhammer, Felix Meurer, Tina Dorosti, Daniel Sasse, Jessica Ritter, Yannik Leonhardt, Franz Pfeiffer, Florian Schaff, Daniela Pfeiffer

**Affiliations:** 1 Department of Diagnostic and Interventional Radiology, TUM School of Medicine and Health, Technical University of Munich, Munich, Germany; 2 Department of Physics, TUM School of Natural Sciences, Technical University of Munich, Munich, Germany; 3 Munich Institute of Biomedical Engineering, Technical University of Munich, Munich, Germany; 4 Institute for Advanced Study, Technical University of Munich, Munich, Germany

**Keywords:** Sparse sampling, Dose reduction, Computed tomography pulmonary angiogram, Convolutional neural network, Pulmonary embolism

## Abstract

This retrospective study evaluates U-Net-based artifact reduction for dose-reduced sparse-sampling CT (SpSCT) in terms of image quality and diagnostic performance using a reader study and automated detection. CT pulmonary angiograms from 89 patients were used to generate SpSCT data with 16 to 512 views. Twenty patients were reserved for a reader study and test set, the remaining 69 were used to train (53) and validate (16) a dual-frame U-Net for artifact reduction. U-Net post-processed images were assessed for image quality, diagnostic performance, and automated pulmonary embolism (PE) detection using the top-performing network from the 2020 RSNA PE detection challenge. Statistical comparisons were made using two-sided Wilcoxon signed-rank and DeLong two-sided tests. Post-processing with the dual-frame U-Net significantly improved image quality in the internal test set, with a structural similarity index of 0.634/0.378/0.234/0.152 for FBP and 0.894/0.892/0.866/0.778 for U-Net at 128/64/32/16 views, respectively. The reader study showed significantly enhanced image quality (3.15 vs. 3.53 for 256 views, 0.00 vs. 2.52 for 32 views), increased diagnostic confidence (0.00 vs. 2.38 for 32 views), and fewer artifacts across all subsets (*P* < 0.05). Diagnostic performance, measured by the Sørensen–Dice coefficient, was significantly better for 64- and 32-view images (0.23 vs. 0.44 and 0.00 vs. 0.09, *P* < 0.05). Automated PE detection was better at fewer views (64 views: 0.77 vs. 0.80, 16 views: 0.59 vs. 0.80), although the differences were not statistically significant. U-Net-based post-processing of SpSCT data significantly enhances image quality and diagnostic performance, supporting substantial dose reduction in CT pulmonary angiography.

## Introduction

Pulmonary embolism (PE) is a common and potentially fatal condition, with an incidence of about 1% in hospitalized patients and a 2% fatality rate among those affected [1, 2].

To confirm or rule out suspected PE in cases with clinical likelihood, computed tomography pulmonary angiography (CTPA) is the gold standard [3]. Nowadays, CTPA has a high diagnostic accuracy [4–6]. However, CTPA comes with the drawback of high radiation exposure, with an average effective dose of 6.1 mSv [7], which is a significant concern due to the risk of malignancy and gametal damage [8]. Approximately 1.5–2.0% of all future cancer cases could be attributed to radiation from CT scans [8, 9]. Therefore, dose reduction is crucial while maintaining suitable image quality, adhering to the principle of “as low as reasonably achievable” (ALARA). Several techniques for dose reduction have been incorporated into clinical practice such as reducing tube voltage or iterative reconstruction algorithms [10–14]. Despite these approaches, dose reduction with conventional CT scanners is limited.

A further dose reduction is possible via new hardware approaches such as sparse sampling CT (SpSCT) [15, 16]. With this technique, fewer projection images can be acquired per rotation. In this method, the radiation beam for each acquired projection is delivered at full tube current, but because the total number of projections is low, the resulting image quality is reduced and streak artifacts appear in the final images. However, the angular sampling decreases simultaneously, degrading the image quality due to an insufficient number of data points. The under-sampled data will result in notable streak artifacts, regardless of the reconstruction algorithm [17].

Machine learning approaches have shown to be suitable for reducing streak artifacts in SpSCT [18–20]. Especially residual learning has shown to deliver superior results. The goal of the network in residual learning is to estimate the difference between SpSCT and full-view images, allowing for the subsequent prediction of artifact-free images. A well-known network architecture for artifact correction is the U-Net [21] and the further developed dual-frame U-Net, which applies artifact correction in the image domain as a post-processing filter [22]. This network proved to be capable of reducing streak artifacts in SpSCT resulting in an increased confidence in detecting lung metastases [23]. Alternative approaches operate in the sinogram domain. Lee et al. proposes an approach to synthesize missing data directly in sparse-view sinograms using deep learning [24]. Hybrid methods address both domains: WNet performs sinogram interpolation and correction, followed by filtered backprojection (FBP) with a learnable kernel and image-domain refinement [25]. Pan et al. propose a multi-domain Swin Transformer network that performs initial recovery, data consistency correction, and high-fidelity reconstruction [26]. Genzel et al. apply corrections in the image domain while incorporating a sinogram-space data fidelity term during training via forward projection [27]. Several works combine deep learning with iterative reconstruction, including the Iterative Residual Optimization Network [28], AirNet [29], and DRONE [30]. While these models improve fidelity to measured data and excellent artifact reduction, their high computational demands hinder clinical applicability. Self-supervised methods based on implicit neural representations [31] and Gaussian splatting [32] eliminate the need for paired datasets but are limited by reconstruction time, resolution constraints, and the need for scan-specific optimization. More recently, diffusion models have been applied to SpSCT, modeling full-dose priors and enabling posterior sampling conditioned on sparse and ultra-sparse sinogram data [33]. Although these methods deliver high-quality reconstructions, their generative nature raises concerns about hallucinations and diagnostic reliability, in addition to high computational cost.

Despite these advances, most studies evaluate results using quantitative image quality metrics, which may fail to reflect the recovery of clinically significant but subtle features [34]. While some works have investigated diagnostic performance in low-dose CT [35], few studies have evaluated the diagnostic impact of sparse sampling with subsequent deep learning-based artifact reduction [19, 23].

Therefore, this study aims to assess the diagnostic impact of U-Net-based artifact reduction in dose-reduced SpSCT. We apply the dual-frame U-Net, an established and accessible method, for artifact reduction. We focus on the specific task of pulmonary embolism (PE) detection, evaluating both automated detection performance and reader-based diagnostic accuracy.

## Methods

### Network Architectures and Training

**Fig. 1:**
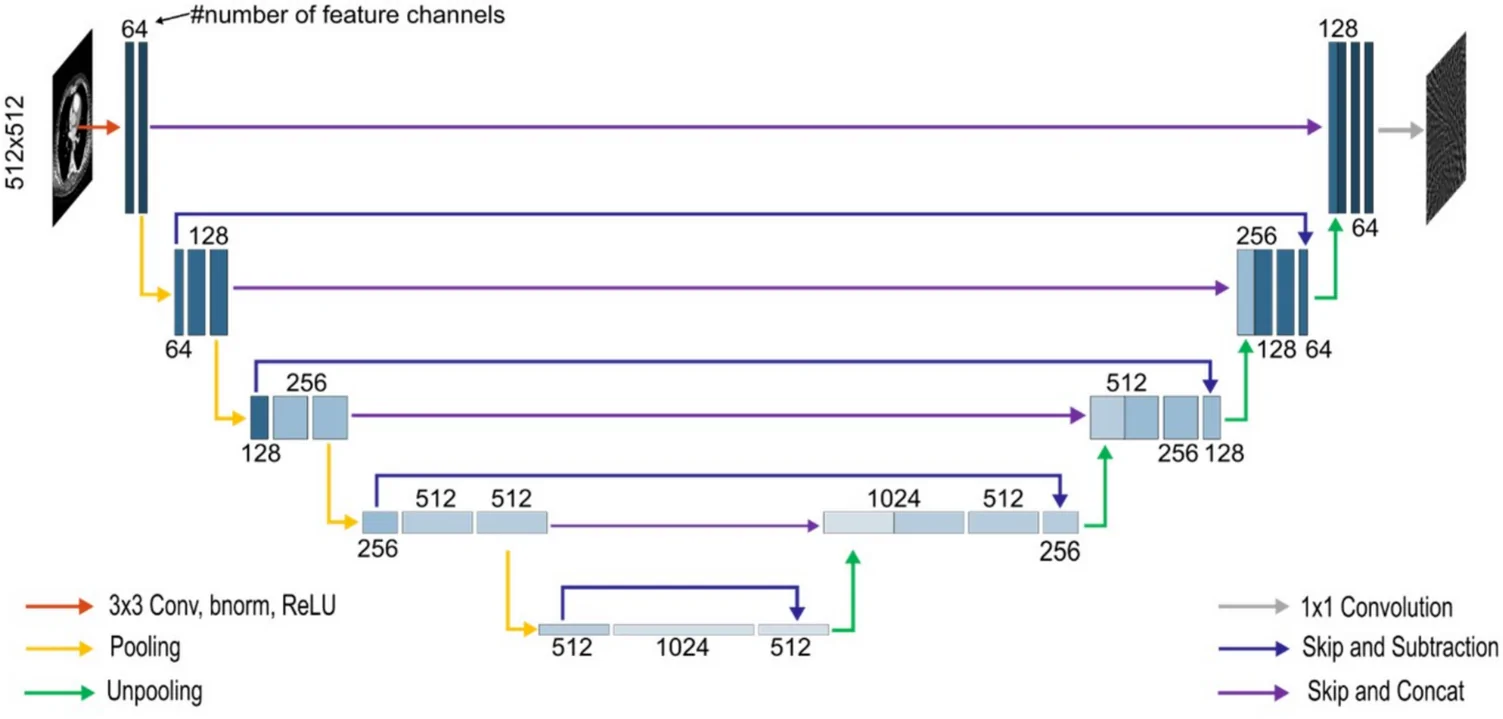
Architecture of the used dual frame U-Net for a 512 × 512 pixel input, adapted from [22]

For artifact reduction, the dual frame U-Net architecture by Han et al., depicted in Fig. 1, was used for its good compromise between performance and computational expense [22]. Its encoder part consists of four subsequently applied decoder blocks, each consisting of two convolutional layers followed by a rectified linear unit (ReLU), respectively. After each encoder block, max pooling is applied. After two convolutional layers in the bottleneck, the features are upsampled again by four subsequently applied decoder blocks, each consisting of, again, two convolutional layers with ReLU. Before each decoder block, unpooling is applied. The convolutional layers use a 3 × 3 convolutional kernel; the pooling and unpooling layers use a 2 × 2 kernel. The output of each encoder block is connected to the input of each decoder block via skip + concatenate connection. The final image is then obtained via convolution by a 1 × 1 kernel. In addition to the standard U-Net, the used dual frame U-Net variant includes an additional skip connection (skip and subtraction) that links the feature maps directly after pooling with those directly before unpooling. This modification satisfies the frame condition which has been shown to improve performance. The network was trained separately for each sparse-view subset, using sparse-view images as input and the difference between full-view and sparse-view images as target. It was trained (keras v.2.11.0) with mean squared error loss for 50 epochs with a mini-batchsize of 16. The initial learning rate was 0.001 and decayed exponentially per epoch following ${\left\{l{r}_{n}=l{r}_{\left\{n-1\right\} e}\right\}}^{\left\{-0.1\right\}}$

For automated PE detection, we utilized the top-performing model from the 2020 RSNA PE detection challenge by Guanshuo Xu, referred to as the PE-detection network [36]. This model comprises two backbone networks (SE-ResNeXt101 and SE-ResNeXt50) for slice-level label prediction and a recurrent neural network that uses the backbone network embeddings to produce exam-level PE predictions [37]. For both, we observed the validation loss to ensure no overfitting occurred and the model with the smallest validation loss among all epochs was chosen.

All models were trained on an Nvidia RTX 3090 with 24 GB VRAM.

### Dataset

**Fig. 2:**
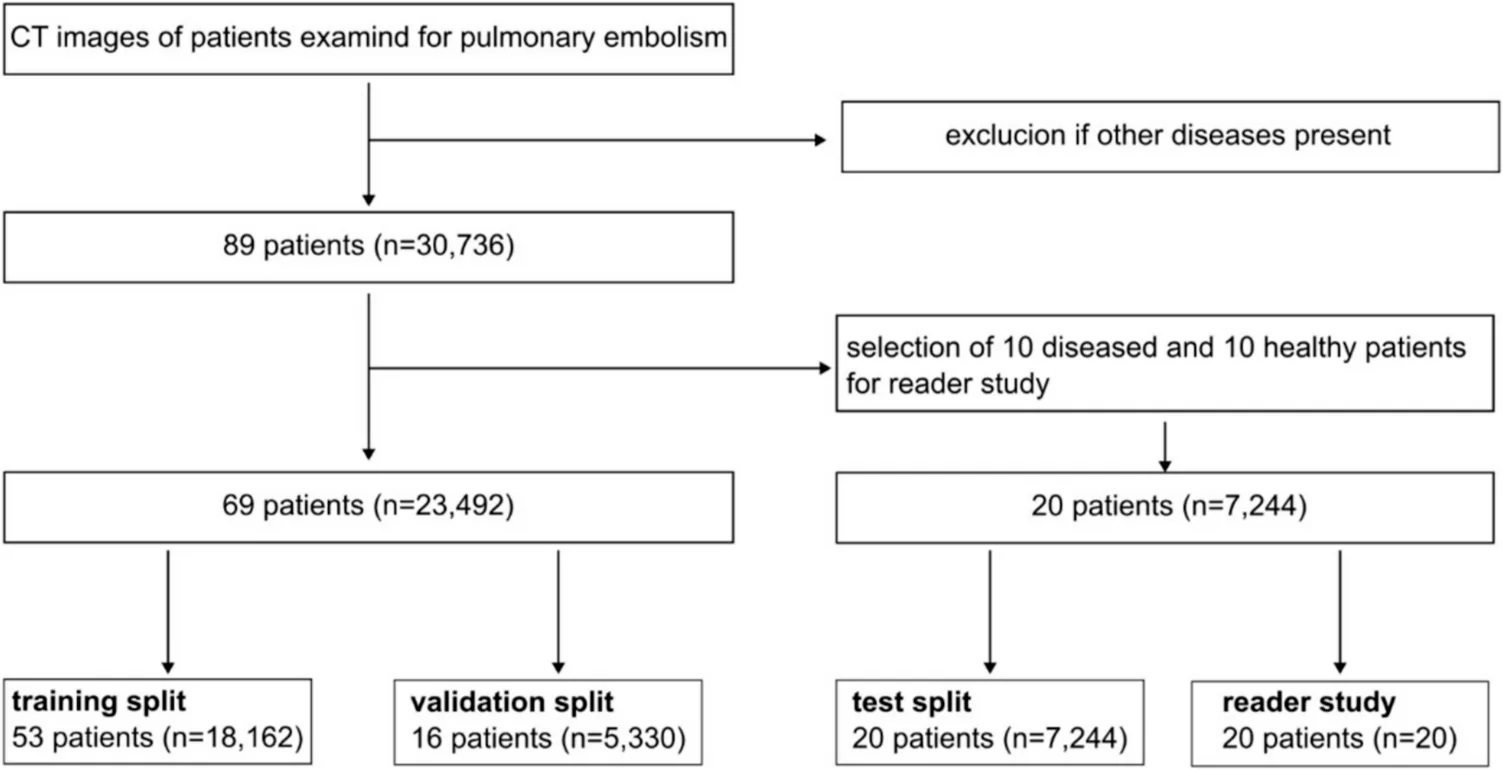
Flowchart of the data selection process. A total of 30,736 CT images from 89 patients examined for pulmonary embolism were retrospectively selected from our clinical data storage and archiving system (*n* = number of CT slices)

The study utilized data collected retrospectively, approved by the institutional review board, from 89 anonymized CTPA scans (60 healthy, 29 with PE) between October 2019 and January 2023. Due to its retrospective nature, informed consent was waived. The study was conducted in accordance with the regulations of our institution. The dataset was divided, keeping 20 patients for a reader study and test set, while the rest were randomly allocated on patient level into training (77%) and validation (23%) groups, as illustrated in Fig. 2. Patient demographics and dose information are detailed in Table 1, with effective dose calculated using the dose-length-product from the CT dose report and a chest conversion factor (0.014), according to (26).

**Table 1:** Patient demographics and dose information for the internal dataset

Characteristics	Train (*n* = 53)	Validation (*n* = 16)		Test/reader study (*n* = 20)
PE cases	15	4	10	
Gender				
Male	20	8	8	
Female	33	8	12	
Age (years)	33.79 (20–43)	35.69 (19–44)	36.0 (26–42)	
Effective dose (mSv)	1.64 (0.93 6.18)	1.74 (1.31, 2.82)	1.84 (0.84, 7.32)	

Age and effective dose are given as mean (range)

*PE *pulmonary embolism

To create sparse-view CTs, sinograms with 2048 views (corresponding to full-view images) were created under parallel beam geometry from the CT dataset using the Astra Toolbox (v2.1.0) ​(27)​. Target images were reconstructed from 2048 views using filtered back projection (FBP). Six sparse-view subsets were generated using FBP with 16, 32, 64, 128, 256 and 512 views, respectively. All images were clipped to the lung window [center: − 600 HU, width: 1700 HU] and normalized to (0, 1).

For external testing, 10,000 images were randomly chosen from the labeled train split of the RSNA pulmonary embolism CT dataset (28)​, pre-processed identical to the internal dataset. The PE-detection model was trained from scratch (torch v.2.0.1) on 7279 labeled CT pulmonary angiograms from the RSNA dataset, following Guanshuo Xu’s methodology (28)(23).

### Quantitative Evaluation

The assessment of image quality for sparse-view images both before and after post-processing was conducted by using the test split of our internal dataset, along with the selected images from the RSNA dataset. This assessment was carried out using two metrics: the structural similarity index measure (SSIM) and the peak signal-to-noise ratio (PSNR). Furthermore, 95% confidence intervals (CIs) were computed for the results obtained.

### Reader Study

In this single-blind study, 20 CT scans were utilized (10 healthy, 10 PE). From each scan, one representative image was selected, sparse sampled using either 256, 128, 64, 32, or 16 views and subsequently post-processed by the trained dual-frame U-Net. The resulting sparse-view images, both with and without post-processing, were then used for the reader study, leading to a total of 200 images. Images reconstructed from 512 views were not included in the reader study due to their similarity in quality to full-view images.

Each image was independently evaluated by three board-certified radiologists with 3, 5, and 5 years of experience, respectively, marking emboli and assessing image quality, diagnostic confidence, and artifact severity according to a predefined scale displayed in Table 2.

**Table 2:** Categories and possible answers used in the reader study

Scale	Quality	Confidence		Artifacts
0	Not diagnostic	Not confident at all		A lot of artifacts and reduced quality
1	Highly impaired	Slightly confident		Some artifacts and reduced quality
2	Impaired	Somewhat confident		Few artifacts and quality not impaired
3	Sufficient	Fairly confident		No artifacts
4	High	Very confident		-
5	Very high	Surely confident		-

The gold standard segmentation used full-view images and was established by consensus among two radiologists with 7 and 9 years of experience, respectively.

The study’s outcomes were evaluated by pooling the responses from the three readers. The mean Sørensen–Dice coefficient (SDC), measuring the overlap between gold standard and reader segmentation, and the mean assigned label with 95% confidence intervals were computed from the positive samples for each sub-sampled dataset with and without post-processing, respectively [38].

Images deemed non-diagnostic by the readers were categorized as false positive (FP) or false negative (FN), respectively. For the remaining images, all positive cases with a SDC score greater than 0 were categorized as true positives (TPs), with rest marked as FNs. The negative cases with no segmentation were assigned as true negatives (TNs), the remaining as FPs. From these categorizations, the study calculated the accuracy, sensitivity, and specificity.

### Statistical Analysis

As a Shapiro–Wilk test showed that the calculated SSIM and PSNR metrics did not follow normal distribution, the two-sided Wilcoxon signed-rank test was performed to check for significant differences (SciPy 1.4.1) [38]. The same methodologies were applied for the evaluation of the assigned image quality, diagnostic confidence, and rated artifacts of the reader study, as well as the calculated SDCs, given their non-normal distribution. Automated PE detection was evaluated using the empirical area under the receiver operating characteristic curve (AUC) values, including 95% CIs, estimated as described by DeLong et al. [39, 40]. Thereby, the algorithmic implementation by Sun and Xu adapted to Python version 3.8.10 was used (35). Statistical differences in AUCs between SpSCT and full-view datasets, as well as between different postprocessing methods, were evaluated using the DeLong two-sided test.

### Comparing Methods

To compare the FBP reconstructions and the network predictions, the SpSCT datasets were also reconstructed using the simultaneous iterative reconstruction technique (SIRT) with 100 iterations and the ordered-subsets simultaneous algebraic reconstruction technique (OSSART) with 50 iterations and a block size of 8. Both methods were implemented using the TIGRE toolbox (version 3.0) and employed a regularization parameter *λ* = 1.

## Results

### Image Quality

**Fig. 3:**
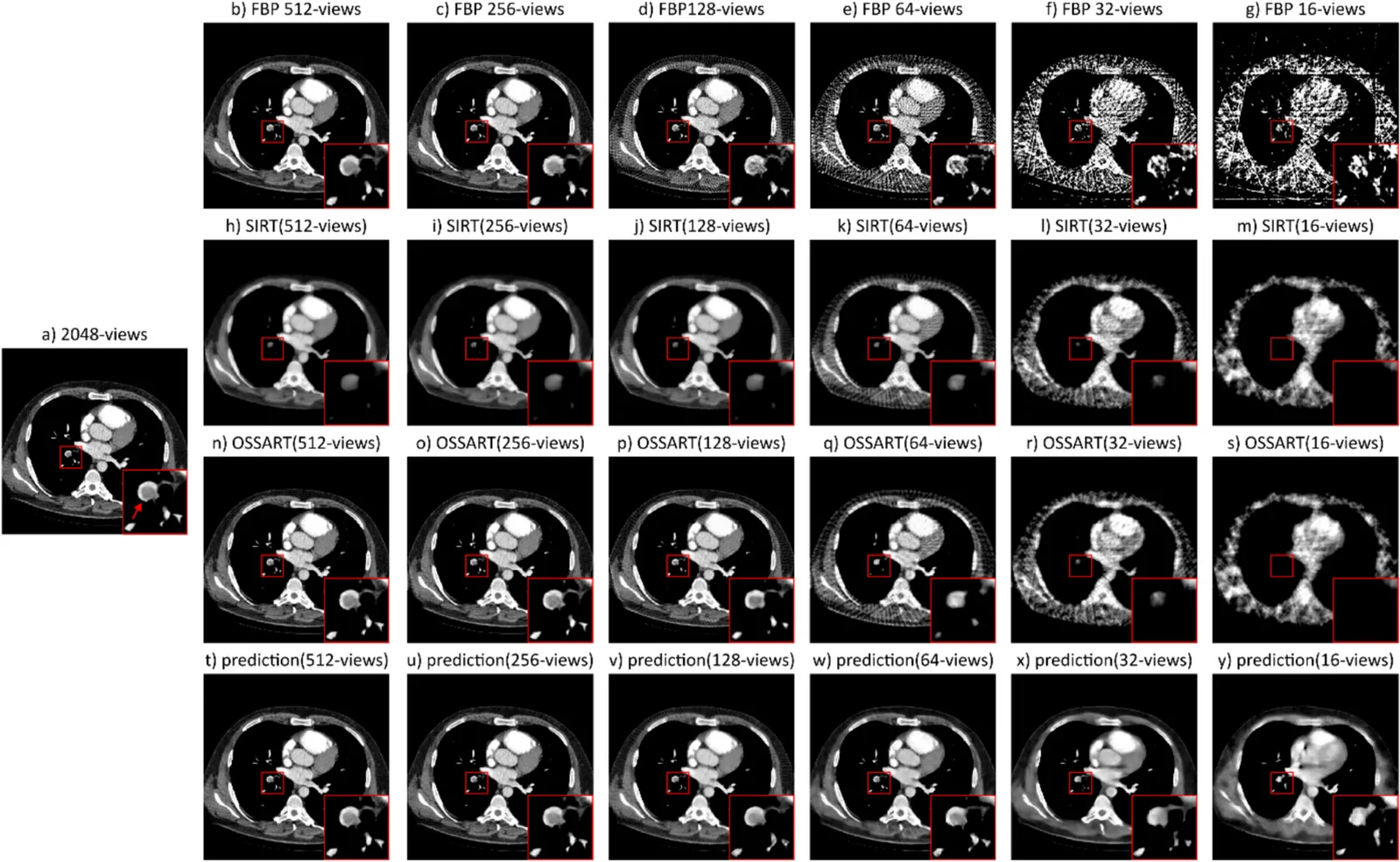
CT image (512 × 512 pixels) from the test set labeled with pulmonary embolism (red arrow). **a** Ground truth image reconstructed via FBP from 2048 views. **b–g** Same image reconstructed via FBP from 512, 256, 128, 64, 32, and 16 views, respectively. The PE and surrounding blood vessels remain recognizable down to 128 views, but by 64 views, artifacts begin to obscure vascular structures, worsening in the 32- and 16-view images. **h–m** Reconstructions using simultaneous iterative reconstruction technique (SIRT) with decreasing views. While SIRT reduces artifacts compared to FBP, the resulting images appear overly smoothed. **n–s** Reconstructions using ordered-subsets simultaneous algebraic reconstruction technique (OSSART) with decreasing views. OSSART reduces artifacts similarly to SIRT but better preserves image features. **t–y** Network predictions of the corresponding images in the first row (**b**–**g**). The network reduces streak artifacts but also introduces smoothing, particularly under severe undersampling. Nevertheless, the results remain visually closest to the full-view reconstruction. All images are presented in the soft tissue window [center: 60 HU, width: 380 HU]. The insert is 60 × 60 pixels

Figure 3 illustrates the effects of varying the number of views in CT image reconstruction. As the number of views decreases, the presence of artifacts increases, significantly distorting lung features in the 32- and 16-view images. This effect is most pronounced in the FBP reconstructions, which exhibit strong streak artifacts. SIRT and OSSART reconstructions reduce these artifacts but introduce oversmoothing. SIRT, in particular, excessively smooths the images, leading to loss of fine features. OSSART preserves more structure, with the pulmonary embolism (PE) remaining discernible down to 128 views. At lower view counts, however, both methods increasingly suppress image details. Post-processing the FBP reconstructed images with the dual-frame U-Net reduces streak artifacts and improves image quality, allowing the PE to remain recognizable down to 64 views, though with a loss of sharpness in image features.

Table 3 presents the mean SSIM and PSNR values obtained from the reconstructed images of the test set and the RSNA dataset, with individual values calculated in reference to the respective 2048-view images reconstructed via FBP. The application of the dual-frame U-Net for post-processing the SpSCT images resulted in a noticeable improvement in PSNR values for all cases in both datasets by reducing the streak artifacts in comparison to raw FBP reconstructions. The observed differences in mean values were significant for all cases (*P* < 0.001). Furthermore, for the internal dataset, dual-frame U-Net post-processing led to a significant improvement in SSIM values across all cases (*P* < 0.001). For the external RSNA dataset, post-processing of the 512-view and 256-view images led to a reduction in the respective SSIM values. For the remaining subsets generated from the RSNA dataset, the post-processing resulted in increased SSIM values. In all cases, SIRT and OSSART reconstructions yield significantly worse PSNR and SSIM values compared to the U-Net predictions (*P* < 0.001). For 128 views and fewer, SIRT achieves higher PSNR and SSIM than FBP. OSSART outperforms FBP in terms of PSNR and SSIM for 256 views and fewer.

**Table 3:** Structural similarity index measurement (SSIM) and peak signal-to-noise-ratio (PSNR) values of the sparse-sampled CT images of the internal test split and the RSNA dataset

Internal dataset test split						
SSIM	512 views	256 views	128 views	64 views	32 views	16 views
FBP	0.989	0.911	0.634	0.378	0.234	0.152
	(0.989–0.989)	(0.91–0.911)	(0.633–0.635)	(0.377–0.379)	(0.233–0.234)	(0.152–0.152)
SIRT	0.873	0.871	0.843	0.740	0.645	0.609
	(0.872–0.873)	(0.871–0.872)	(0.842–0.843)	(0.739–0.741)	(0.644–0.646)	(0.608–0.61)
OSSART	0.976	0.952	0.846	0.708	0.632	0.608
	(0.976–0.976)	(0.951–0.952)	(0.846–0.847)	(0.707–0.709)	(0.631–0.634)	(0.606–0.609)
U-Net	0.997	0.981	0.894	0.892	0.866	0.778
	(0.997–0.997)	(0.981–0.981)	(0.894–0.895)	(0.891–0.893)	(0.866–0.867)	(0.776–0.779)
PSNR[dB]	512 views	256 views	128 views	64 views	32 views	16 views
FBP	41.271	31.558	26.406	21.389	17.028	13.788
	(41.257–41.285)	(31.544–31.572)	(26.387–26.424)	(21.364–21.414)	(17.005–17.051)	(13.766–13.809)
SIRT	26.927	26.927	26.775	25.764	23.601	21.291
	(26.893–26.962)	(26.892–26.961)	(26.741–26.809)	(25.733–25.794)	(23.574–23.627)	(21.267–21.315)
OSSART	34.26	32.707	29.965	26.648	23.696	21.261
	(34.115–34.406)	(32.608–32.805)	(29.913–30.017)	(26.615–26.68)	(23.669–23.722)	(21.237–21.285)
U-Net	53.725	45.297	35.847	35.227	32.457	27.865
	(53.683–53.766)	(45.257–45.337)	(35.815–35.878)	(35.191–35.264)	(32.417–32.497)	(27.827–27.903)
RSNA dataset						
SSIM	512 views	256 views	128 views	64 views	32 views	16 views
FBP	0.988	0.941	0.699	0.429	0.279	0.198
	(0.988–0.988)	(0.941–0.941)	(0.698–0.699)	(0.428–0.429)	(0.279–0.28)	(0.197–0.198)
U-Net	0.957	0.927	0.787	0.755	0.699	0.616
	(0.957–0.958)	(0.926–0.928)	(0.786–0.789)	(0.753, 0.756)	(0.697–0.701)	(0.614–0.618)
PSNR[dB]	512 views	256 views	128 views	64 views	32 views	16 views
FBP	40.65	34.519	29.197	23.514	18.638	14.912
	(40.637–40.662)	(34.492–34.546)	(29.17–29.224)	(23.491–23.536)	(18.618–18.658)	(14.893–14.93)
U-Net	46.664	40.428	30.618	29.052	25.599	21.467
	(46.559–46.77)	(40.34–40.517)	(30.543–30.693)	(28.976–29.129)	(25.531–25.668)	(21.408–21.527)

The images of the internal test split were reconstructed by either filtered back projection (FBP), simultaneous iterative reconstruction technique (SIRT), or ordered-subsets simultaneous algebraic reconstruction technique (OSSART) from varying number of views. In addition, the FBP reconstructions were post-processed by the U-Net. The images of the RSNA test split were reconstructed via FBP and post-processed by the U-Net. The data are presented as means with 95% CI in parentheses

*SSIM *structural similarity index measurement, *PSNR *peak signal-to-noise-ratio, *FBP *filtered back projection, *CI *confidence interval

### Reader Study

**Fig. 4:**
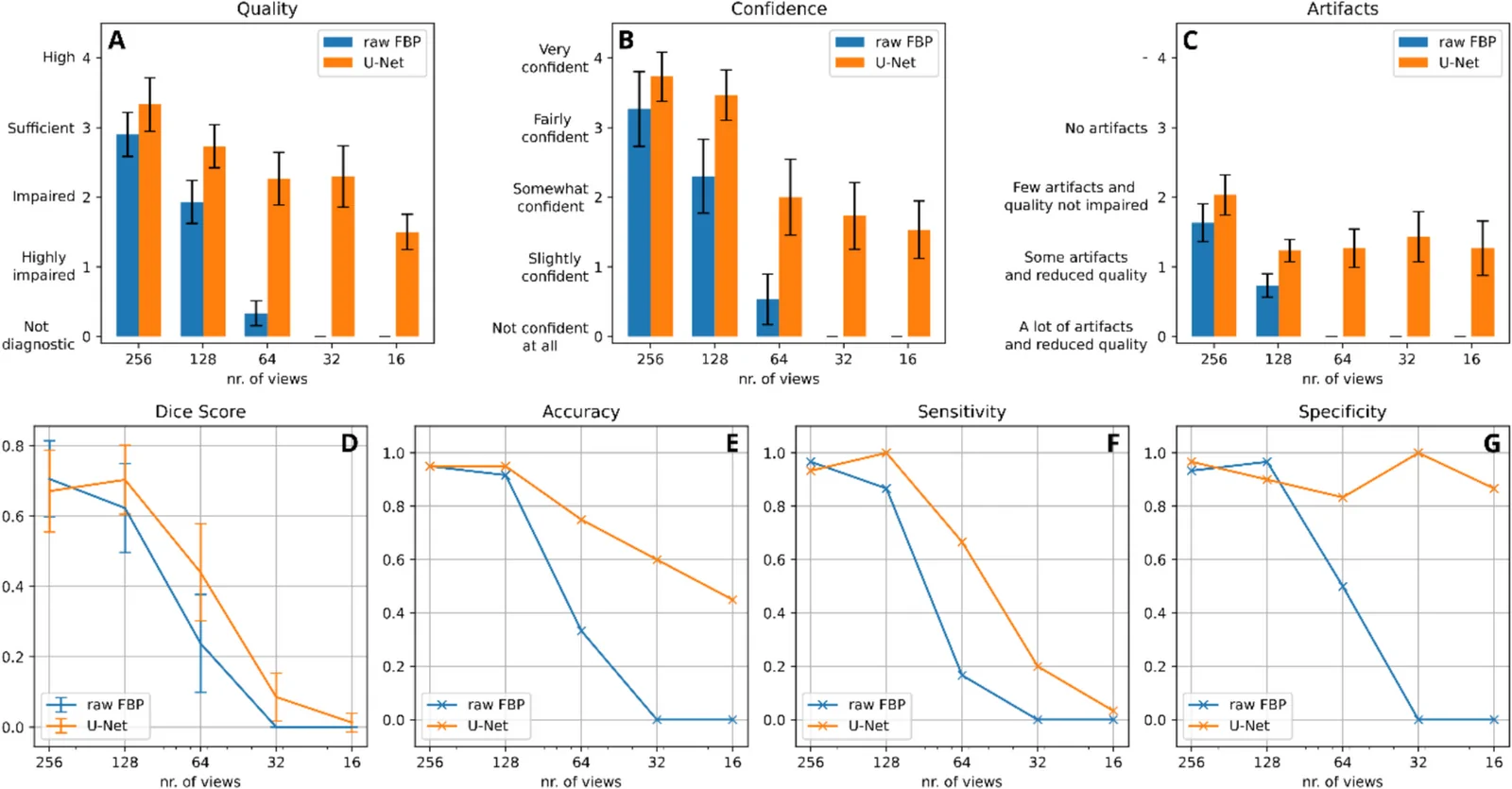
Results of the reader study.** A**–**C** Mean of the assigned quality, diagnostic confidence, and artifact assessment of the 20 sparse-view images labeled by the three readers without (FBP) and with post-processing by the dual-frame U-Net (U-Net) with 95% confidence intervals. The labels associated with the scales are given in Table 2. **D** Results of the segmentation task of the reader study in terms of mean Sørensen–Dice coefficient (SDC) with 95% confidence intervals. **E**–**G** Accuracy, sensitivity, and specificity of all the readers combined with respect to the number of views used for reconstruction, without post-processing (FBP) and with dual-frame U-Net (U-Net) post-processing

Figure 4a–c presents the means of the reader-assigned values for quality, diagnostic confidence, and artifact assessment. Across all levels of subsampling, a declining image quality is observed with decreasing reconstruction views. In the case of raw sparse-view images, the mean quality rating decreases linearly from *sufficient* [3.15; 95% CI: (2.94–3.36)] for the 256-view images to *not diagnostic* [0.42 (0.29–0.55)] for the 64-view image and further diminish to 0.00 (0.00–0.00) for the 32-view and 16-view images, respectively. With dual frame U-Net post-processing, a higher image quality is noted for every subset with a smaller decrease in subjective image quality for decreasing views. Here, image quality ranged from *high* [3.53 (3.28–3.79)] for 256 views to *impaired* [2.23 (2.00–2.47)] for 64 views. The 32-view images were rated as *sufficient* [2.52 (2.21–2.82)] and the 16-view images as *impaired* [1.65 (1.40–1.89)]. For all levels of subsampling, the rated image quality for dual frame U-Net post-processing significantly surpasses that of raw images (*P* < 0.01).

Similar trends are found in the diagnostic confidence assessment. For raw reconstructions, confidence decreases from *very confident* [3.5 (3.21–3.79)] for the 256-view reconstruction to not confident at all [0.00 (0.00–0.00)] for the 32-view case. With post-processing, rated diagnostic confidence remains at *very confident* for 256 views, decreasing to *somewhat confident* [2.38 (1.84–2.52)] for the 32-view subset and [1.89 (1.55–2.22)] for the 16-view subset. Across all subsampling levels, the reported diagnostic confidence of post-processed images is higher (*P* < 0.01).

Regarding perceived artifacts, both 256-view image subsets are rated as *few artifacts and quality not impaired* [1.87 (1.71–2.03) and 2.11 (1.93–2.30), respectively]. Raw reconstructions exhibit an increase in artifacts with decreasing number of views to *a lot of artifacts and reduced quality* for the 64-view reconstructions [0.00 (0.00–0.00)]. With artifact reduction by the dual frame U-Net, the image rating maintains a rating of *some artifacts and reduced quality* or higher, even for 16-view reconstructions [1.28 (1.01–1.56)]. Across all subsampling levels, the reported presence of artifacts is reduced for the post-processed images (*P* < 0.01).

Figure 4d presents the mean SDCs of the segmentation task. The SDC values acquired from both the 256-view and 128-view reconstructions, with and without dual frame U-Net post-processing, exhibit no statistical differences (*P* > 0.05). In the case of 64-view reconstructions, a decline in SDC values is discernible with a significantly higher value for post-processed images (0.23 vs. 0.44; *P* = 0.004). For 32 views, the mean SDC reduces to 0.00 for raw reconstructions and to 0.09 for the post-processed images (*P* = 0.02). The mean SDCs of the 16-view images with and without post-processing exhibit no significant difference (*P* = 0.32).

The accuracy, sensitivity, and specificity of the readers across the different subsets are displayed in Fig. 4e–g, while the corresponding confusion matrices are shown in Table 4. In terms of accuracy and sensitivity, a downward trend is noticeable as the number of reconstruction views decreases, both for images with and without post-processing; hereby, the decline is smaller for post-processed images, especially for the 64-view and 32-view reconstructions. The specificity of the raw FBPs also decreases with decreasing number of views. Meanwhile, the specificity of post-processed images shows a decrease down to the 64-view reconstructions, followed by an increase and, subsequently, another decrease. This is because, for the 32- and 16 view images, readers produced fewer segmentations overall, which led to fewer instances of false negative cases, resulting in higher specificity. However, this reduced segmentation also results in fewer true positive cases and subsequently, lower sensitivity. All raw FBP cases reconstructed from 32 or 16 views were labeled as “not diagnostic” by the readers, resulting in automatic classification as false positive or false negative, respectively.

**Table 4:** Confusion matrices of the reader study for the three readers with varying levels of subsampling without post-processing (FBP) and with dual-frame U-Net (U-Net) post-processing

Nr. of views		256		128		64		32		16	
	Actual	Yes	No	Yes	No	Yes	No	Yes	No	Yes	No
	Rating										
FBP	Yes	29	2	26	1	9	0	0	0	0	1
	No	1	28	4	29	21	30	30	30	30	29
U-Net	Yes	28	1	30	3	21	5	7	0	1	0
	No	2	29	0	27	9	25	23	30	29	30

*FBP *filtered back projection

**Fig. 5:**
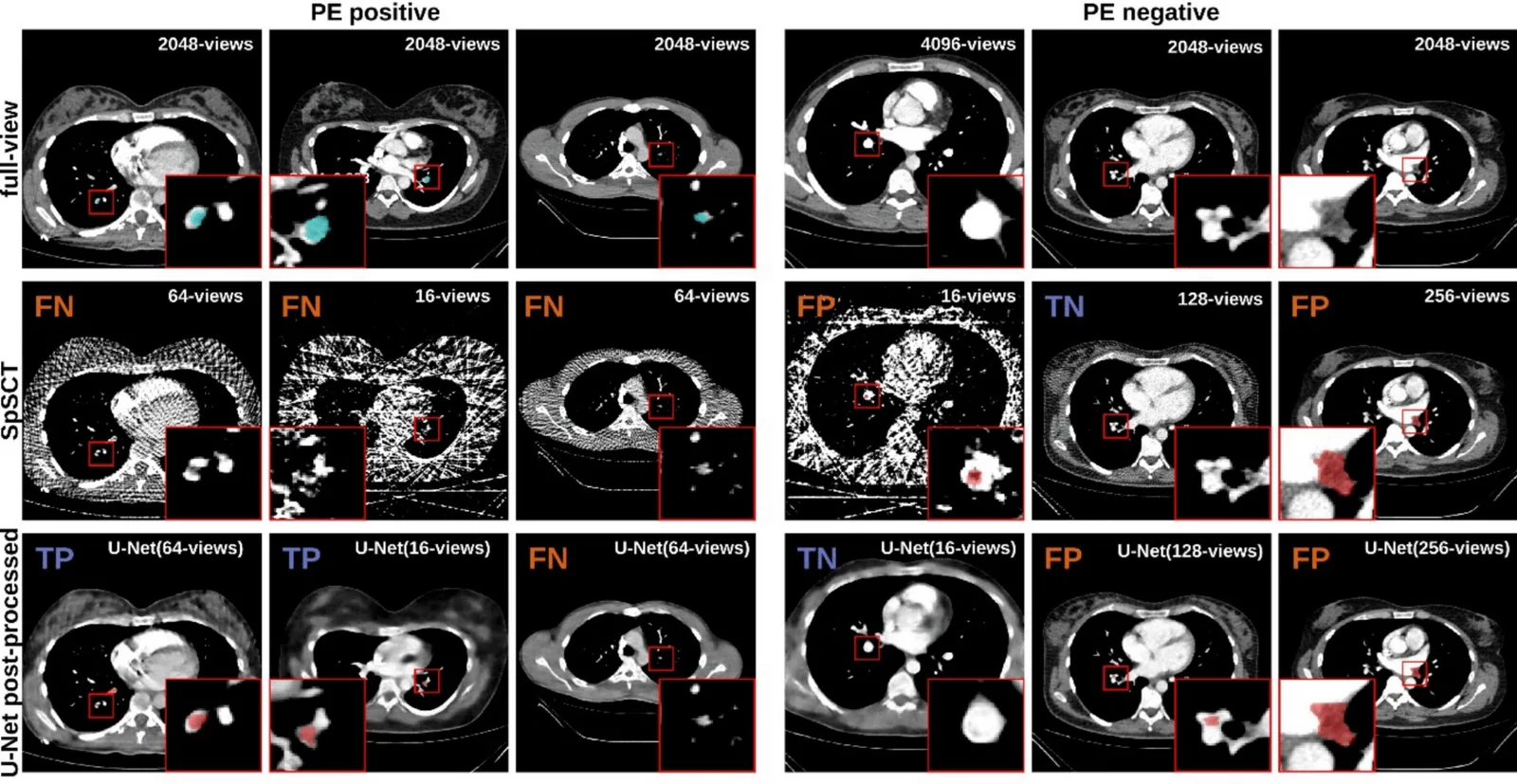
Examples of pulmonary embolism (PE) segmentations. Each column displays a full-view image, followed by its corresponding SpSCT (sparse sampling CT) reconstruction, and then the U-Net post-processed SpSCT image. The first three columns depict images labeled with PE, while the last three columns show images labeled as healthy. The gold standard segmentations are shown in the PE positive full-view images as blue; the segmentations by the readers in red. In the upper left corner of each image, it is indicated whether the images were correctly marked (true positive [TP] or true negative [TN]) or not (false negative [FN] or false positive [FP]). All images are presented in the soft tissue window [center: 60 HU, width: 360 HU]

Figure 5 presents examples of images included in the reader study, showcasing both the reader’s segmentations and the corresponding full-view reconstructions alongside gold standard segmentations. In the first example not only the original embolism was marked but also a healthy vessel. As the PE was found nonetheless, this was counted as TP.

### Automated PE Detection

**Fig. 6:**
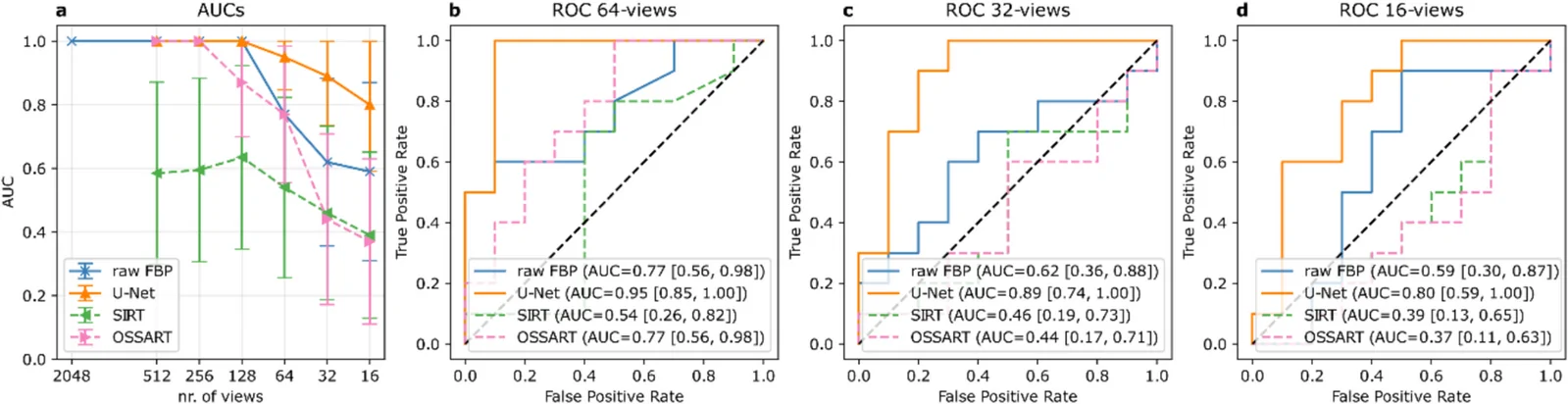
Exam-wise results of the automated PE detection. **a** Graphs depict the mean area under the receiver operator characteristic curve (AUC) values of the sparse sampling CT (SpSCT) datasets without and with U-Net postprocessing as well as the simultaneous iterative reconstruction technique (SIRT) and ordered-subsets simultaneous algebraic reconstruction technique (OSSART) reconstructions. 95% confidence intervals (CIs) are indicated by the error bars around each data point. **b–d** Individual receiver operator characteristic (ROC) curves for the 64-view, 32-view, and 16-view cases, respectively

Figure 6 illustrates the performance of the PE-detection network on the FBP reconstructions with and without U-Net post-processing, the SIRT, and the OSSART reconstructions. The detection model achieved perfect AUC scores down to 128-view subsampling for the FBP reconstructions with and without U-Net post-processing. For the 64-view FBP reconstructions, the AUC dropped to 0.77 [95% CI: 0.56, 0.98] for raw images and further decreased to 0.59 [95% CI: 0.30, 0.87] for 16-view reconstructions. With U-Net post-processing, detection performance remained higher, with an AUC of 0.95 [95% CI: 0.85, 0.98] for the 64-view case and 0.80 [95% CI: 0.59, 1.00] for the 16-view reconstructions. A comparison of AUCs with and without U-Net post-processing, using the DeLong method, yielded *p*-values of 0.09, 0.08, and 0.27 for the 64-view, 32-view, and 16-view subsets, respectively. With SIRT reconstruction, the AUC is 0.59 [95% CI: 0.30, 0.87] for 512 views, decreasing to 0.39 [95% CI: 0.13, 0.65] for 16 views. In contrast, OSSART achieves perfect AUC scores for 512- and 256-view reconstructions. The AUC declines to 0.87 [95% CI: 0.70, 1.00] at 128 views and further drops to 0.37 [95% CI: 0.11, 0.63] for 16 views. When comparing the AUCs of the SIRT reconstructions to those of the U-Net post-processed images, differences are significant (*P* ≤ 0.01) across all view counts, except for the 16-view case, where *P* = 0.02. AUC comparisons between OSSART and the U-Net outputs yield *p*-values of 0.1, 0.06, 0.003, and 0.01 for the 128-, 64-, 32-, and 16-view cases, respectively, indicating significant differences in performance for the latter two subsets.

## Discussion

In this study, we explored the potential of the dual-frame U-Net for artifact reduction in SpSCT and its subsequent impact on PE detection, assessing both reader performance and automated detection accuracy. Our results demonstrate that post-processing SpSCT scans with the dual-frame U-Net substantially improves visual image quality. Quantitative evaluation of our internal test split, based on SSIM and PSNR values, confirmed these enhancements. However, for the external RSNA dataset, post-processing the 512-view and 256-view images resulted in a decrease in SSIM values. This trend may be attributed to the network’s tendency to replicate the data distribution of the ground truth training images from our internal dataset, leading to reduced structural similarity with the full-view images from the RSNA dataset, which originated from a different source. Furthermore, we assessed the diagnostic value of SpSCT, both with and without U-Net post-processing, through a reader study. The results showed that U-Net post-processing significantly improved the SDC when using 64 or 32 views for reconstruction. Across all subsets examined, the reported image quality, diagnostic confidence, and reduction in image artifacts were significantly enhanced following U-Net post-processing. Additionally, our findings suggest an improvement in automated PE detection after U-Net post-processing for SpSCTs with 64 or fewer views, although this improvement was not statistically significant in our study. Even though our training data had an uneven distribution of positive and negative PE cases, we could not observe that this imbalance impacted the image quality of the PE positive cases or the subsequent detection tasks. Since the network is trained in a residual manner to detect sparse-sampling artifacts rather than directly reconstruct images, it focuses on the structure of artifacts rather than specific image features (e.g., PE presence). This makes it more robust and less dependent on case-specific content.

We compared U-Net post-processing of FBP reconstructions with two iterative reconstruction methods: SIRT and OSSART. While both iterative methods effectively reduce undersampling artifacts, they introduce substantial oversmoothing, particularly in the case of SIRT. U-Net post-processing yielded significantly better quantitative performance in terms of PSNR and SSIM. It also improved automated pulmonary embolism (PE) detection, with significantly higher AUCs compared to SIRT. While U-Net post-processing also outperformed OSSART in PE detection, the statistical evidence for this comparison was less strong. We hypothesize that the oversmoothing introduced by the iterative methods may impair the performance of the PE detection network by suppressing subtle features necessary for accurate detection. Additionally, the public dataset used to train the PE detection model differs from the SIRT and OSSART reconstructions, potentially causing a domain shift and further degrading detection performance due to out-of-distribution inputs. Compared with deep learning–based methods from literature, the dual-frame U-Net by Han et al. shows a substantial improvement in PSNR from 22.28 to 38.79 dB for 60 views [22]. Similarly, WNet improves PSNR from 26.13 ± 1.31 to 37.10 ± 1.35 dB at 128 views, yielding results comparable to those observed in our study [25]. More advanced architectures, such as the multi-domain integrative Swin transformer (43.64 dB at 64 views) [34] and AirNet (25.2 dB to 54.5 dB at 60 views) [29], report notably higher PSNR values. This is expected, as both methods are significantly more complex and leverage information from both the image and sinogram domains. However, direct comparison of PSNR across studies must be approached with caution, as each employs different training, validation, and test splits. Moreover, standard image metrics like PSNR and SSIM often poorly reflect perceived image quality and diagnostic relevance, as shown by Verdun et al. [41], highlighting the need for clinical evaluation alongside numerical metrics.

The current study demonstrates the potential of combining two emerging technologies—sparse sampling as a hardware solution on the one hand and machine learning as a software technology on the other hand. As SpSCT comes with the drawback of increasing streak artifacts at lower doses and fewer views, machine learning–based image optimization is an ideal completion of this technology. For both approaches individually, and especially for machine learning–based image optimization, the potential of improving image quality was shown previously [15, 23, 42, 43]. Han et al. showed the potential of U-Net variants to improve image quality in SpSCT [22]. In the named study, real CT data of patients were used to demonstrate the potential of the method. However, no diagnosis had to be made, and neither a reader study nor an automated lesion detection was performed. As for both SpSCT and machine learning algorithms, loss of image information is feared; it is important to prove the preserved diagnostic validity. We could show that radiologists could detect even subsegmental pulmonary embolisms with a high accuracy in SpSCT images obtained with fewer views and that the cutoff is clearly better when images were post-processed using dual-frame U-Net.

Automated or assisted detection of pulmonary embolism is one of the many emerging applications of artificial intelligence in radiology. Multiple studies showed that automated PE detection has a high accuracy and can thus accelerate the workflow in clinical practice, e.g., by highlighting examinations with a suspected PE [44, 45]. Thus, it is important that those algorithms can work with post-processed low-dose images as well. In our study, the algorithm for automated PE detection works sufficiently in those low-dose datasets with a clear improvement by U-Net post-processing, which is in accordance with the reader study.

Assuming a linear relationship between the number of views and radiation dose, our results suggest that a dose reduction to approximately 6.3% of the original level (128/2048 views), corresponding to a mean effective dose of 0.11 mSv, is achievable using SpSCT. At this level, a very high accuracy is present even for images without post-processing; no significant difference can be found in post-processed data. For the lower dose levels (64 views and fewer), there is a significant difference between data with and without post-processing. However, as the accuracy drops even in post-processed images, this cannot be recommended for clinical practice. Once SpSCT, in combination with post-processing, is used in a routine setting, one has to find the optimal number of views and estimate the actual radiation dose, either through direct measurements or more sophisticated dose simulation software. Nonetheless, our findings indicate that greater dose reduction is possible using post-processing.

This study has several limitations that should be considered. The SpSCT data were retrospectively generated from CT volumes under simplified conditions, which may not accurately represent real-world applications. However, as SpSCT scanners are not yet available, these generated images had to be used. Additionally, readers were provided with only one slice per CT volume and a fixed window setting, which is not in accordance with clinical practice. Third, FBP was used as the reconstruction method. Nowadays, iterative reconstruction algorithms are used. However, as FBP was used for images with and without U-Net post-processing, this seems sufficient for this feasibility study. Lastly, with only 89 CT volumes, the dataset is relatively small compared to other CT datasets, which makes it more difficult to evaluate the significance of some results. Increasing the amount of training data could potentially improve the model’s performance. In addition, no clinically viable hardware solution for sparse-view CT is currently available.

In conclusion, post-processing of CT-images derived from a SpSCT with reduced projections can lead to a significant dose reduction in CTPA, surpassing what is achievable with sparse sampling alone. The obtained images deliver a high diagnostic value for radiologists as well as for automated diagnosing.

## Data Availability

The models and the data are available upon reasonable request to the corresponding author Johannes Thalhammer (johannes.thalhammer@tum.de).

## Abbreviations

ALARA: As low as reasonably achievable
AUC: Area under the receiver operator characteristic
CI: Confidence interval
CTPA: Computed tomography pulmonary angiography
FBP: Filtered back projection
FN: False negative
FP: False positive
HU: Hounsfield unit
PE: Pulmonary embolism
PSNR: Peak signal-to-noise ratio
SDC: Sørensen–Dice coefficient
SpSCT: Sparse sampling computed tomography
SSIM: Structural similarity index measure
TN: True negative
TP: True positive
